# Assessment of Anterior Cingulate Cortex (ACC) and Left Cerebellar Metabolism in Asperger's Syndrome with Proton Magnetic Resonance Spectroscopy (MRS)

**DOI:** 10.1371/journal.pone.0169288

**Published:** 2017-01-06

**Authors:** Aya Goji, Hiromichi Ito, Kenji Mori, Masafumi Harada, Sonoka Hisaoka, Yoshihiro Toda, Tatsuo Mori, Yoko Abe, Masahito Miyazaki, Shoji Kagami

**Affiliations:** 1 Department of Pediatrics, Institute of Biomedical Sciences, Tokushima University Graduate School, Tokushima, Japan; 2 Department of Child Health & Nursing, Institute of Biomedical Sciences, Tokushima University Graduate School, Tokushima, Japan; 3 Department of Radiology, Institute of Biomedical Sciences, Tokushima University Graduate School, Tokushima, Japan; University of New Mexico, UNITED STATES

## Abstract

**Purpose:**

Proton magnetic resonance spectroscopy (^1^H MRS) is a noninvasive neuroimaging method to quantify biochemical metabolites *in vivo* and it can serve as a powerful tool to monitor neurobiochemical profiles in the brain. Asperger’s syndrome (AS) is a type of autism spectrum disorder, which is characterized by impaired social skills and restrictive, repetitive patterns of interest and activities, while intellectual levels and language skills are relatively preserved. Despite clinical aspects have been well-characterized, neurometabolic profiling in the brain of AS remains to be clear. The present study used proton magnetic resonance spectroscopy (^1^H MRS) to investigate whether pediatric AS is associated with measurable neurometabolic abnormalities that can contribute new information on the neurobiological underpinnings of the disorder.

**Methods:**

Study participants consisted of 34 children with AS (2–12 years old; mean age 5.2 (±2.0); 28 boys) and 19 typically developed children (2–11 years old; mean age 5.6 (±2.6); 12 boys) who served as the normal control group. The ^1^H MRS data were obtained from two regions of interest: the anterior cingulate cortex (ACC) and left cerebellum.

**Results:**

In the ACC, levels of N-acetylaspartate (NAA), total creatine (tCr), total choline-containing compounds (tCho) and myo-Inositol (mI) were significantly decreased in children with AS compared to controls. On the other hand, no significant group differences in any of the metabolites were found in the left cerebellum. Neither age nor sex accounted for the metabolic findings in the regions.

**Conclusion:**

The finding of decreased levels of NAA, tCr, tCho, and mI in the ACC but not in left cerebellar voxels in the AS, suggests a lower ACC neuronal density in the present AS cohort compared to controls.

## Introduction

Asperger’s syndrome (AS) is a brain disorder characterized by impaired social skills and nonverbal communication, as well as restrictive and repetitive patterns of behavior and activities [1]. Although AS has been reclassified as an autistic spectrum disorder (ASD) in the 5^th^ edition of the Diagnostic and Statistical Manual of Mental Disorders (DSM-5) [2], there have been suggestions that AS and ASD can present with distinct verbal styles, motor signs, emotion perception, and pragmatic reasoning [3].

Pathophysiologically, a number of limbic and cortical structures are believed to be implicated in AS/ASD [4]. Due to the ritualistic behavior and impaired social interactions in AS/ASD, a great deal of attention has been focused on the anterior cingulate cortex (ACC), as the structure in the limbic system involved in response inhibition, in delineating between the perception of self and others, as well as in error detection, all of which are impaired in AS/ASD [5]. Further implicating the ACC are the results of task-related functional magnetic resonance imaging (fMRI) studies, which have consistently found ACC hypoactivation in ASD [6–9].

In the present study, we sought to advance our understanding of the neurobiological underpinnings of AS by using proton magnetic resonance spectroscopy (^1^H MRS), a noninvasive neuroimaging technique that enables *in vivo* examination of brain metabolism and chemistry, to investigate potential neurometabolic abnormalities in the ACC and cerebellum of children with AS.

Several prior ^1^H MRS studies in ASD [10,11], and three in AS have been reported [12–14]. A meta-analysis of ^1^H MRS data from the ACC of children with ASD found significant decreases in the levels of the putative neuronal marker, N-acetylaspartate (NAA), compared to controls [10]. By contrast, levels of frontal lobe NAA were found to be higher in AS than in controls in the two prior studies [12,13]. Despite these discrepant NAA findings in ASD and AS, we hypothesized that, as in the two prior studies in AS, NAA would be increased in the disorder compared to controls, while no differences would be observed in the cerebellum. In secondary analyses, levels of total creatine (tCr), total choline (tCho), myo-inositol (mI), combined glutamate and glutamine–referred to as Glx–and γ-aminobutyric acid (GABA) were compared between the groups.

## Methods

### Participants

For this study, which was approved by the Institutional Review Board of Tokushima University, 34 children (2–12 years old; mean age: 5.2 ± 2.0; 28 boys) diagnosed with AS according DSM-IV-TR criteria [1], were recruited from among the outpatients of the Department of Pediatrics. To participate in the study, each child’s parent or legal guardian provided written informed consent according to the principles of Declaration of Helsinki. Children old enough to understand the content and purpose of the study also provided their assent.

Nineteen children (2–11 years old; mean age: 5.6 ± 2.6; 12 boys) referred for MRI examination due to a non-specific temporal symptom (e.g., headache or vertigo), but were otherwise healthy, were recruited to serve as the normal comparison group (Table 1). To minimize anxiety and motion, all participants were sedated with 0.5ml/kg body weight of triclofos sodium, which was administered approximately one hour before the MRI scan and monitored according to the sedation guidelines of the American Academy of Pediatrics [15]. We first examined ^1^H MRS in the ACC followed by the left cerebellum. For children who could not lie still we only measured ^1^H MRS in the ACC.

**Table 1:** Participant Characteristics and Demographics.

		N	Gender(Boy/girl)	Age(year)	Age(mean±SD)
ACC	AS	34	28/6	2–12	5.2±2.0
	Control	19	12/7	2–11	5.6±2.6
Left cerebellum	AS	23	19/4	4–9	5.1±1.4
	Control	12	9/3	2–12	6.5±2.8

AS, Asperger’s syndrome

### ^1^H MRS measurement

All neuroimaging studies were conducted on a General Electric 3.0 T Signa HD MRI system (Milwaukee, WI, USA) with a standard volume birdcage radiofrequency head coil.

**Fig 1:**
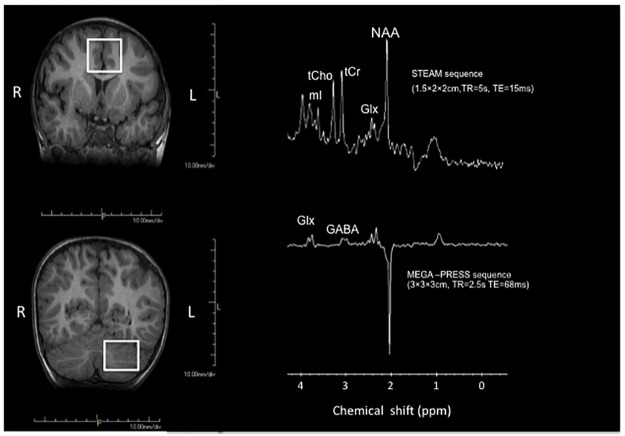
Positions of the measurement voxel in ACC (anterior cingulate cortex) and left cerebellum and representative spectra from each voxel. (LEFT) Positions of the measurement voxel in the anterior cingulate cortex (ACC) and left cerebellum. (RIGHT) Representative short TE and J-edited spectra obtained.

To acquire the brain ^1^H MRS data, two methods were implemented. First, conventional short echo time (TE) spectra were obtained using the Stimulated Echo Acquisition Mode (STEAM) sequence with repetition time (TR) = 5000 ms, echo time (TE) = 15 ms and 48 signal averages to record spectra from a 1.5×2.0×2.0-cm^3^ voxel prescribed in the ACC and in the left cerebellum (Fig 1). Next, without moving the subjects spectra were again obtained from a 3x3x3-cm^3^ voxel, but using the standard Point RESolved Spectroscopy (PRESS) sequence, which had been modified to enable the detection of γ-aminobutyric acid (GABA) by J-edited spin echo difference technique [16], as fully described recently [17,18]. Briefly, to implement the J-editing technique, a pair of a frequency-selective inversion pulses was inserted into the standard PRESS method, and then applied on the GABA C-3 resonance at 1.9 ppm on alternate scans, using TE/TR 68/2500ms. This resulted in two subspectra in which the GABA C-4 resonance at 3.03 ppm and Glx C-2 resonance at 3.71 ppm were alternately inverted or not inverted. Subtracting the two subspectra yielded a spectrum consisting only of the edited GABA C-4 and Glx C-2 resonances, with all overlapping resonances eliminated. For each voxel, the data were acquired in 10.1 min using 128 interleaved excitations (256 total), with the editing pulse on or off. The magnetic field homogeneity for the acquisitions was typically ≤12Hz, as assessed from the full width at half maximum of the unsuppressed voxel tissue water resonance.

#### ^1^H MRS data processing and quantification

Both the short-TE PRESS and J-edited spectral data were processed according now established methods and then quantified with the LCModel package (V. 6.2), using a basis set of MR spectra experimentally measured from a phantom containing GABA, glutamine, glutamate, NAA tCr, tCho, and myo-inositol, with default LCModel macromolecule (MM) resonances at or near 0.9, 1.2, 1.4, 1.7 and 2.0 ppm. All the metabolites were quantified as “absolute” concentrations in mmol/L by LCModel using the unsuppressed water signal in each voxel as a signal intensity and concentration reference, assuming a water content of 82% [19]. The reliability of the LCModel-derived metabolite levels was expressed with Cramer-Rao lower bounds (CRLB), with a relative CRLB (%SD) of 20% set as the upper cutoff limit of acceptable uncertainty for each metabolite. By this standard, no spectra were rejected due to a high quantification uncertainty.

### Statistical analysis

Mean levels of the metabolites of interest were compared between children with AS and controls. In primary analyses, NAA alone was compared between the groups, and in secondary, exploratory analyses, tCr, tCho, mI, Glx and GABA were compared, without correction for multiple comparisons. Student’s t-tests were used to assess the differences between AS and controls, and each group’s gender differences at the significance of p = 0.05 (two-tailed). Pearson’s correlation coefficient was used to assess for potential associations between age and metabolite levels in AS.

## Results

Levels of ACC NAA, tCr, tCho, and mI were lower in children with AS than in the controls (p<0.05). On the other hand, no differences of these metabolites were found in the cerebellum (Table 2). Furthermore, no significant differences in any of the metabolites were found between boys and girls either within the AS or within control groups and no significant associations were found between age and any of the metabolite levels in AS.

**Table 2:** Metabolite concentrations (mmol/L).

		NAA	tCr	tCho	mI	Glx	GABA
ACC	AS(n = 34)	5.6±0.8*	4.6±0.7**	1.2±0.2*	3.5±0.9**	9.1±2.1	0.7±0.3
	control(n = 19)	6.0±0.8	5.2±0.9	1.3±0.3	4.3±1.0	10.0±3.1	0.9±0.4
Left cerebellum	AS(n = 23)	5.9±0.8	5.7±1.1	1.3±0.5	4.1±0.7	8.5±1.5	1.5±0.5
	control(n = 12)	6.1±0.9	5.9±1.0	1.4±0.5	4.6±1.7	8.0±1.6	1.5±0.6

Values are the mean±SD.

*p<0.05;

**p<0.01

ACC anterior cingulate cortex, AS, Asperger’s syndrome. NAA, N-acetylaspartate; tCr, creatine/phosphocreatine; tCho, choline-containing compounds; mI, myo-Inositol; Glu, glutamate; Gln, Glutamin; Glx, Glu+Gln. GABA, gamma-aminobutyric acid.

## Discussion

In this study, we found levels of NAA in the ACC to be significantly lower in children with AS than in age- and sex-matched control children, while no such difference was found in the cerebellum. In secondary analyses, we also found lower levels of tCr, tCho, and mI in the ACC of children with AS compared to controls, but no differences in the cerebellum. These results are in disagreement with the results of two prior studies in adults with AS, which reported elevations of ACC and frontal lobe NAA compared to controls [12,14]. A third prior study had measured levels of metabolites in amygdala-hippocampal complex in 10–50 years old AS, and found no significant difference compared to controls although it reported report a significant age-related reduction in NAA, NAA/Cr, and tCho in the AS group [13], suggesting potential differences in these metabolites between adults and children (vide infra). With respect to the levels of the other metabolites, one of the prior studies, reported elevations of prefrontal tCr and tCho [12], which were also in disagreement with the results of the present study.

While the reason for the discrepancy between the present results of decreased ACC and frontal neurometabolites in AS and prior studies that reported elevations of the same metabolites in similar brain regions could be methodological or due to differences in AS cohort characteristics, the difference in age may be the primary reason for the discrepancy: in the present study we have investigated only children with AS where prior studies had investigated only adults [12,13] or a mixture of children and adults [14]. In fact, to our knowledge, the present study is the first to use ^1^H MRS to assess the neurometabolic characteristics of children with AS, and age-dependent metabolic differences have been reported, albeit in different regions [10, 13].

Interestingly, a meta-analysis of ^1^H MRS data in ASD, which included AS, had found NAA to be decreased specifically in whole-brain gray and while matter in children [10]. In addition, and importantly, the meta-analysis showed age-dependent changes in parietal cortex, the cerebellum, and the anterior cingulate cortex [10]. These findings suggest metabolite levels in AS may fluctuate with age, although we found no significant association between age and levels of any of the metabolites, likely due to the limited age range and sample size of this study.

The involvement of the ACC in the executive function neuronal network and its role in the cognitive control attention are well established [20,21]. In addition, the ACC has close anatomic connections to the amygdala and the orbitofrontal cortex, which are participants in emotional expression. In non-human primates, experimentally-induced ACC lesions lead to poor vocal and facial expression, with tendency toward isolation from and poor communication with other primates in the colony [22]. In humans, likewise, injury to the ACC leads to decreased social interactions and increased isolation, and decreased verbal communication, although with increased tendency to interact with and manipulate inanimate objects [22]. Our finding of decreased ACC NAA in children with AS is consistent with abnormal ACC function in the disorder.

The meta-analysis of ^1^H MRS studies in ASD suggested a strong association between the degree of NAA abnormalities and developmental changes, especially in frontal lobe. ASD children with larger-than-normal brain size also had lower-than-normal NAA levels, suggesting that early increase in brain size in children with ASD may occur through an increase in non-neuron tissues, such as glial cell proliferation [10].

As with the levels of NAA, we found levels of tCr, tCho and mI levels to be significantly lower in the ACC in children with AS than in controls, but not in the cerebellum. This selective reduction in metabolite levels in the ACC region but not in the cerebellum strongly suggests a decrease in the density and/or function of neuronal and glia cells density in the ACC of children with AS. Such cellular abnormalities in a key brain regulatory structure can plausibly contribute to difficulty in modulating emotional reactivity that characterizes children with AS and ASD.

This study has two of notable limitations. First, the sample size was relatively small and clinical records for several patients were incomplete, which limited statistical power and the ability to detect potential associations between clinical variables, including AS severity scores, and demographic variables vs. metabolite levels. Second, we did not obtain volumetric MR images to assess potential tissue volume differences and to correct the metabolite levels for tissue heterogeneity within the voxels of interest. However, no abnormal signal or atrophy was detected in the brain of AS or controls. The findings of this study should therefore be interpreted taking into consideration these caveats.

In summary, this study has found that NAA and the other major brain metabolites detectable by ^1^H MRS are significantly lower in the ACC of children with AS than in matched healthy controls, which provides additional evidence implicating ACC is implicated in the pathophysiology of AS. If replicated, the results of this study could lead to the development of ^1^H MRS as a noninvasive technique for characterizing metabolic abnormalities in children with AS that could serve as diagnostic and therapeutic response biomarkers for the disorder.

## Supporting Information

S1 Dataset The file summarizes all the relevant data that have been used in the statistical analyses. (XLSX)
